# IronDeficiency Across Neurodevelopmental Disorders: Comparative Insights from ADHD and Autism Spectrum Disorder

**DOI:** 10.3390/children13020180

**Published:** 2026-01-28

**Authors:** Lourdes M. DelRosso, Lilliana Estrada Chaverri, Fernando Alberto Ceballos Fuentes

**Affiliations:** 1 Department of Medicine, University of California, San Francisco–Fresno, Fresno, CA 93701, USA; 2 SleepMed, Medical Center for Diagnosis and Treatment of Sleep Disorders, San José 10201, Costa Rica; l.estrada@sleepmedcostarica.com; 3 Neurology & Sleep Center, Guatemala City 01010, Guatemala; neurologoguatemala@gmail.com

**Keywords:** iron deficiency, neurodevelopmental disorders, attention-deficit/hyperactivity disorder

## Abstract

**Highlights:**

**What are the main findings?**

• In children with ADHD, neuroimaging studies show reduced brain iron in key dopaminergic regions, although peripheral iron markers and their association with ADHD-related symptoms are inconsistent.
• In autism spectrum disorder, low ferritin is consistently associated with sleep-related motor disturbances, and iron supplementation improves sleep outcomes.

**What are the implication of the main finding?**

• Evaluation of iron status should be considered in children with ADHD and ASD, particularly in those with sleep disturbances, motor restlessness, or suboptimal response to standard treatments.
• Iron supplementation, when guided by laboratory monitoring, may serve as a useful adjunctive therapy to improve sleep and selected functional outcomes.

**Abstract:**

**Background:** Iron plays a crucial role in neurotransmitter synthesis, myelination, and neuronal metabolism. Iron deficiency has been associated with a variety of neurodevelopmental disorders, particularly attention-deficit/hyperactivity disorder (ADHD) and autism spectrum disorder (ASD). However, the prevalence, clinical impact, and treatment implications differ between these conditions. **Objective:** To synthesize current evidence on the prevalence, neurobehavioral consequences, and therapeutic implications of iron deficiency in ADHD and ASD, highlighting convergences and disorder-specific findings. **Results:** In ADHD, studies using serum ferritin and related peripheral markers show inconsistent associations with core symptom severity, with reported ferritin thresholds for deficiency ranging widely. While some studies suggest links between low ferritin and hyperactivity, inattention, or stimulant response, others report null findings. In contrast, emerging neuroimaging evidence consistently demonstrates reduced brain iron in dopaminergic regions in children. In ASD, the strongest link is between low ferritin and sleep-related motor disturbances, and iron supplementation may improve sleep and motor symptoms. **Conclusions:** Screening for iron status and targeted supplementation may improve sleep and behavioral outcomes in ADHD and ASD, meriting integration into clinical practice and further randomized controlled trials.

## 1. Introduction

Iron is a critical micronutrient for neurodevelopment, playing an essential role in neuronal energy metabolism, myelination, synaptogenesis, and neurotransmitter synthesis, particularly within dopaminergic and glutamatergic pathways that regulate arousal, motor control, and sleep–wake function [1]. Neurodevelopment is a dynamic, age-dependent process characterized by coordinated changes in brain structure, function, and metabolism across the lifespan. Neuroimaging studies have shown changes in brain structure, function, and metabolism across the lifespan. In 1990, Van Der Knaap et al. [2] used magnetic resonance spectroscopy to show that brain metabolism changed in children aged 3 to 16 years. Glutamine increases during the first years of childhood and then decreases from childhood to adulthood, reflecting maturational changes in the function or the density of glial cells [3]. Equally, brain volume peaks at ages of 10.5 years in females and 14.5 years in males, following an inverse-U-shaped trajectory with age [4]. Gray matter volume also shows an inverted U-shaped trajectory, while the white matter volume increases through adolescence, secondary to an increase in myelin [4]. These changes in volume can affect movements during sleep, as demonstrated by Ferri et al. [5] showing that the development of periodic leg movements mirrors changes in dopaminergic networks, not shared by the other leg movement indices. Synaptic density also increases significantly to a maximum during mid-childhood, and then declines during adolescence [6]. Regional cerebral blood flow increases during infancy and into school age, then decreases to adult levels around age 16–18 years [7]. These developmental processes are closely linked to iron status, particularly to dopaminergic neurotransmitter synthesis (via iron-dependent tyrosine hydroxylase), synaptic pruning, myelination, and neuronal energy metabolism which is supported by mitochondrial function. Iron serves as a cofactor for enzymes involved in the synthesis of dopamine, norepinephrine, and serotonin, as well as in mitochondrial metabolism and myelination [8]. Iron-dependent enzymes such as tyrosine hydroxylase, phenylalanine hydroxylase, and tryptophan hydroxylase catalyze the formation of catecholamines that regulate attention, mood, movement, and circadian rhythms [9]. Iron deficiency, particularly during sensitive developmental windows, has been shown to alter neuronal excitability, synaptic pruning, and dopaminergic signaling, all of which are integral to the pathophysiology of attention-deficit/hyperactivity disorder (ADHD) and autism spectrum disorder (ASD) [10]. However, in addition to iron, processes such as cortical maturation are also shaped by dynamic environmental factors, including social, cognitive, and stress-related experiences that modulate neurodevelopmental trajectories. Recent work integrating structural and functional neuroimaging has demonstrated that environmental exposures, including peer contexts, can influence cortical architecture, reward circuits, and psychopathology-related outcomes during adolescence, illustrating a multilevel model in which iron-dependent neurobiological mechanisms interact with environmental modifiers to influence neurodevelopment and behavioral phenotypes relevant to ADHD and ASD [11]. Furthermore, motor and cognitive circuits undergo substantial plasticity during neurodevelopment, enabling sensorimotor networks to reorganize and compensate through experience-dependent mechanisms. Beyond synaptic remodeling, activity-dependent myelination and white matter plasticity contribute to motor learning and behavioral adaptation [12], providing a framework for understanding how disruptions in sleep or iron-dependent dopaminergic pathways may contribute to motor phenotypes such as restless legs syndrome (RLS) and periodic limb movements of sleep (PLMS) [13].

Dietary iron is absorbed in the duodenum and regulated primarily by hepcidin, a liver-derived hormone that controls intestinal absorption and macrophage iron release in response to iron stores, inflammation, and erythropoietic demand [14]. Children are particularly vulnerable to iron deficiency due to rapid brain and somatic growth, increased iron requirements during infancy and adolescence, limited dietary intake of bioavailable iron, and common feeding patterns that include high milk consumption and selective eating [15]. As a result, iron deficiency frequently occurs in children without overt anemia and may remain unrecognized unless specifically screened for using appropriate biomarkers. Serum ferritin is the most widely used indicator of body iron stores; however, it is an acute-phase reactant and may be elevated in the presence of inflammation, infection, or obesity, potentially obscuring iron deficiency [16,17]. Recent work highlights the clinical relevance of non-anemic iron deficiency (NAID), defined as biochemical evidence of iron deficiency in the absence of anemia. NAID has been associated with neuropsychiatric and cognitive symptoms, including fatigue, anxiety, attention difficulties, and reduced quality of life, suggesting that symptomatology may emerge prior to hematologic compromise [18]. Across studies included in the recent systematic review, NAID was operationalized using ferritin and transferrin saturation thresholds that varied widely, with ferritin cutoffs ranging from 12 to 50 ng/mL, underscoring the lack of consensus regarding optimal diagnostic criteria [18]. Importantly, benefits from iron supplementation have been noted in the presence of biochemical iron deficiency rather than anemia, and neurobehavioral symptoms may be iron-responsive even when hemoglobin remains within the normal range [19].

Serum iron represents circulating iron bound to transferrin but is highly variable due to diurnal fluctuation and recent dietary intake, limiting its utility as a standalone marker [16]. Transferrin and transferrin saturation reflect iron transport and tissue availability; low transferrin saturation indicates inadequate iron delivery to cells [17]. Soluble transferrin receptor (sTfR) reflects cellular iron demand and erythropoietic activity and is relatively unaffected by inflammation, making it a valuable adjunct marker when ferritin interpretation is uncertain [16].

Despite accumulating evidence linking iron deficiency to ADHD and ASD, the literature remains fragmented across nutrition, psychiatry, and sleep medicine disciplines. Heterogeneity in diagnostic thresholds, supplementation regimens, and outcome measures has hindered consensus on screening and treatment protocols. This review, therefore, aims to synthesize data on the prevalence and pathophysiologic mechanisms of iron deficiency in ADHD and ASD; examine its behavioral and sleep consequences; and evaluate therapeutic outcomes from iron supplementation trials. By integrating findings across these domains, this paper seeks to clarify whether iron deficiency represents a shared, modifiable biological substrate across neurodevelopmental disorders and to inform clinical strategies for early detection and intervention.

## 2. Methodology

### 2.1. Literature Search Strategy

A literature search was conducted in PubMed/MEDLINE and EMBASE to identify studies examining the relationship between iron deficiency and attention-deficit/hyperactivity disorder (ADHD) and autism spectrum disorder (ASD). The search strategy combined Medical Subject Headings (MeSH) and free-text terms and included the following keywords: “ADHD” OR “attention-deficit/hyperactivity disorder” OR “autism” OR “autism spectrum disorder” AND “iron deficiency.” Searches were limited to human studies published in English.

### 2.2. Study Selection and Eligibility Criteria

Represented original research, observational, or interventional studies were included.

**Figure 1:**
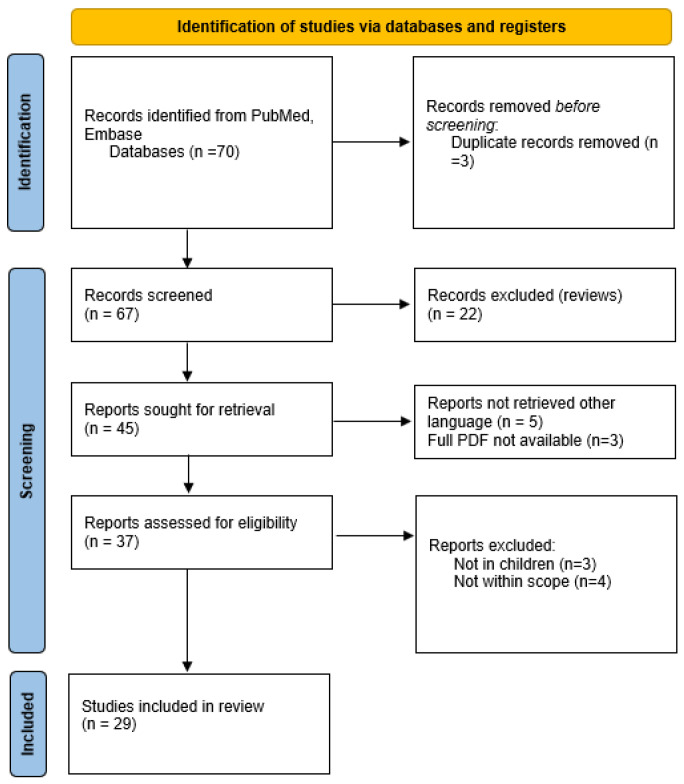
PRISMA diagram of study selection criteria.

Studies were excluded if they investigated iron only as a secondary modifier (environmental metal mixture studies where iron was not the primary exposure), or were non-human studies, or case series of less than 5 subjects (see Figure 1).

### 2.3. Quality Assessment

The search yielded 29 studies. 17 on ADHD and 12 on autism spectrum disorder. The NIH quality assessment tool was used to evaluate the studies (Supplementary Table S1)

Study quality was evaluated using the National Institutes of Health (NIH) Study Quality Assessment Tools, applied systematically to all included studies. Overall, the evidence base was of moderate to good methodological quality, with randomized controlled trials and longitudinal cohort studies [20,21,22,23,24]. Consistently rated as Good due to clearly defined research questions, well-characterized study populations, reliable exposure and outcome measurements, and appropriate statistical analyses. Large population-based studies and mechanistic neuroimaging investigations also demonstrated strong internal validity, despite limited clinical phenotyping in some cases.

In contrast, cross-sectional, case–control, and open-label studies were generally rated as Fair, reflecting inherent design limitations such as the absence of temporal inference, lack of randomization or blinding, small sample sizes, and incomplete adjustment for potential confounders. Across these studies, iron status was frequently assessed using standardized laboratory measures; however, definitions of iron deficiency varied, and many investigations relied on single-time-point ferritin measurements without complementary functional iron markers or longitudinal follow-up. Attrition and exposure misclassification were generally low, but several studies lacked formal justification for sample size or blinded outcome assessment.

Importantly, no studies were rated as Poor, and the consistency of findings across diverse populations, methodologies, and outcome domains supports the overall robustness of the literature. Nonetheless, heterogeneity in study design, definitions of iron deficiency, and outcome measures underscores the need for well-powered prospective trials and longitudinal studies that integrate peripheral and central iron biomarkers with standardized neurobehavioral and sleep outcomes.

## 3. Iron Deficiency in ADHD

### 3.1. Definition 

Studies vary widely in how iron deficiency is defined, contributing to substantial heterogeneity in the reported prevalence. Most studies relied on serum ferritin as the primary marker of iron stores, but used markedly different cutoffs, ranging from <7 ng/mL, corresponding to the assay’s lower limit [20], to <15 µg/L [21], <20 µg/L or composite criteria incorporating additional markers [22], and <30 ng/mL, the threshold most commonly used to define non-anemic iron deficiency [25]. Other large population-based analyses treated ferritin as a continuous variable without categorical cutoffs and adjusted for inflammation using C-reactive protein, thereby precluding prevalence estimates based on iron-deficiency thresholds [26,27]. Beyond ferritin, several studies incorporated other markers of iron status with equally heterogeneous definitions. Hemoglobin was used either to exclude anemia or to infer iron deficiency, with thresholds ranging from <11.7 g/dL in school-aged children [21], to mean baseline values of approximately 9.7–9.9 g/dL in sleep-disturbed, iron-deficient ADHD subgroups [28]. While other trials specifically enrolled children with normal hemoglobin despite low ferritin [25]. Mean corpuscular volume was used to identify microcytosis, with values ≤ 80 fL considered abnormal [29]. Soluble transferrin receptor was employed as a marker of tissue iron deficiency, with an upper limit of >8.3 mg/L used to define abnormality in iron-deficient pediatric cohorts [22]. Zinc protoporphyrin, reflecting impaired heme synthesis, was used as a functional indicator of iron deficiency with thresholds > 70 µmol/mol heme [22]. Transferrin concentrations were occasionally included as part of broader iron profiling but were generally interpreted relative to laboratory reference ranges rather than fixed diagnostic thresholds [25].

Studies in neuroimaging provide a deeper understanding of the iron status in the central nervous system of children with ADHD. A study by Adisetiyo et al. used advanced MRI techniques, including magnetic field correlation (MFC) and relaxometry (R2, R2*, R2′), to assess brain iron indices in children and adolescents with ADHD compared with typically developing controls [30]. This work found that medication-naïve ADHD patients had significantly lower striatal and thalamic brain iron indexes than control subjects, and that these differences were attenuated in psychostimulant-medicated patients, suggesting both a brain iron deficit and modulation by treatment [30]. Importantly, no significant differences were observed in conventional serum measures, underlining a central rather than systemic iron finding. Cascone et al. used MRI-based quantitative susceptibility mapping to estimate brain tissue iron in the basal ganglia and thalamus, regions critical for dopaminergic neurotransmission, and reported atypical relationships between striatal iron and response inhibition performance in both ADHD and typically developing children [31]. Although group means did not differ significantly, children with ADHD showed stronger coupling between putamen iron and methylphenidate (MPH) responsiveness, suggesting that functional iron deficiency within dopaminergic circuits may occur even in the absence of systemic iron deficiency.

Across studies, brain iron was estimated using MRI relaxometry (R2*, R2, magnetic field correlation) and quantitative susceptibility mapping (QSM), with a focus on basal ganglia and thalamic regions, showed that medication-naïve children with ADHD consistently showed lower brain iron concentration compared with neurotypical controls, regardless of imaging modality, scanner type, or population ethnicity [32]. Using quantitative susceptibility mapping MRI, Chen et al. demonstrated region-specific brain iron deficiency in children with ADHD, with iron levels in the anterior cingulate correlating with symptom severity, despite inconsistent findings using peripheral iron markers [33].

### 3.2. Prevalence

The prevalence of iron deficiency in children with ADHD varies depending on population, diagnostic criteria, and ferritin thresholds. In a randomized controlled trial that included 52 children from Thailand, nearly 45% of children with ADHD met criteria for iron deficiency, defined by ferritin < 30 ng/mL; however, this was no different from the general population prevalence of iron deficiency in Thailand (52%) [23]. Similarly, studies have found no differences in serum ferritin or other iron parameters between children with ADHD and controls [34,35]. Calarge et al. studied 52 children with ADHD to assess if baseline iron stores predicted response to treatment. This study found that 87% of children with ADHD had ferritin levels below 30 ng/mL, and 23% of children aged 6–14 had ferritin levels below 7 ng/mL, the laboratory threshold for ID, despite normal hemoglobin [20]. Lahat et al. observed that more than half of children with ADHD had ferritin levels below 20 ng/mL, but only a very weak, clinically insignificant correlation was found between ferritin and ADHD symptom severity [36]. In a large randomized trial of school-aged children with ADHD (n = 502), iron deficiency, defined as ferritin < 15 µg/L was present in 21.7% of participants, a prevalence more comparable to that reported in some pediatric general population samples [21].

### 3.3. Consequences of Iron Deficiency in ADHD

In children with ADHD, some studies showed that iron deficiency has been associated with greater core symptom severity, particularly inattention and hyperactivity, and parent- and teacher-rated ADHD symptom scores, as well as sleep-related motor symptoms, even in the absence of anemia [25,27]. Doom et al. followed Chilean infants with early iron deficiency into adolescence and found greater rates of internalizing, externalizing, and attention-related problems, even after controlling for socioeconomic and perinatal variables [24]. Early deficiency, especially between 6 and 18 months, was linked to increased risk of ADHD-like symptoms later in life [24]. Calarge et al. demonstrated that ferritin concentration correlated inversely with baseline inattention, hyperactivity/impulsivity, and total ADHD scores (r = −0.31 to −0.43, *p* < 0.05), implying that iron-deficient children exhibit greater behavioral dysregulation [20]. Importantly, sleep-related manifestations are prominent, including iron deficiency and symptoms such as restless legs, increased nocturnal motor activity, and sleep fragmentation, which in turn may exacerbate daytime behavioral symptoms [37]. In fact, children with ADHD and a family history of restless legs syndrome had higher severity of symptoms of ADHD [38]. Studies examining functional iron deficiency, defined using ferritin and complementary markers such as zinc protoporphyrin or soluble transferrin receptor, have shown that iron deficiency is associated with greater teacher-rated hyperactivity and attentional difficulties [22]. However, other contributors such as co-existent lead exposure and other nutritional deficiencies, can be confounders [21].

Beyond core symptoms, lower ferritin has been associated with functional differences in stimulant response. In risperidone-treated youth, Calarge et al. reported a mean ferritin of 37 µg/L with 21% below 20 µg/L, despite adequate dietary intake [39]. Low ferritin correlated inversely with disruptive behaviors and weight gain, implying that psychotropic regimens may exacerbate or interact with latent deficiencies [39]. Moreover, withdrawal of methylphenidate has been shown to decrease ferritin levels, suggesting bidirectional relationships between stimulant exposure and systemic iron homeostasis, although ferritin changes did not correlate with symptom worsening, and no participants met criteria for iron deficiency [40]. The authors propose that changes in ferritin observed after stimulant discontinuation may reflect state-dependent effects of medication on iron metabolism or utilization, rather than iron deficiency [40].

However, other studies have not found strong associations between ferritin and ADHD symptoms [35]. In the GINIplus and LISAplus birth cohorts, Romanos et al. examined over 2800 children. They found no cross-sectional or longitudinal association between peripheral ferritin concentrations and parent-reported hyperactivity/inattention scores, including analyses linking ferritin measured at 4 months of age to ADHD symptoms at 10 years [26]. Notably, these analyses modeled ferritin as a continuous variable across the population and did not specifically target iron-deficient children or clinically diagnosed ADHD samples, nor did they assess functional iron deficiency using complementary biomarkers. Similarly, other large observational studies have reported null associations when iron status is examined outside of clinically iron-deficient or symptomatic subgroups, suggesting that ferritin may act less as a general risk factor for ADHD and more as a modifier of symptom severity, sleep-related motor manifestations, or treatment response in vulnerable children [21,22].

### 3.4. Treatment and Therapeutic Implications

Interventional studies of iron supplementation in children with ADHD and iron deficiency suggest potential benefits. In a randomized, placebo-controlled trial of non-anemic children with ADHD and low ferritin (≤30 ng/mL), treatment with oral ferrous sulfate 80 mg/day for 12 weeks resulted in significant improvement in ADHD Rating Scale scores and global clinical severity compared with placebo [25]. Subsequent controlled studies reinforced these findings. In Thailand, Pongpitakdamrong et al. demonstrated that children with iron deficiency receiving ferrous fumarate (200 mg per capsule, equivalent to 66.5 mg elemental iron) at a dose of 2–4 mg/kg/day elemental iron for 12 weeks plus methylphenidate exhibited greater parent-rated improvement on Vanderbilt scales than those receiving methylphenidate alone (mean change –3.96 ± 6.79 vs. 0, *p* = 0.037) [23]. Although teacher ratings showed no difference, the parental perspective, often more sensitive to home behavior, suggests additive benefits of iron repletion.

Combination and adjunctive approaches have also emerged. Yehuda et al. demonstrated that polyunsaturated fatty acid therapy improved concentration and hemoglobin in iron-deficient ADHD children, highlighting synergistic roles of fatty acids and iron in neuronal membrane and dopaminergic integrity [28].

## 4. Iron Deficiency in Patients with Autism Spectrum

### 4.1. Definition

Similarly to the studies in children with ADHD, studies in children with Autism Spectrum Disorders, characterized iron deficiency using various markers, though with somewhat greater emphasis on hematologic indices in addition to ferritin. Serum ferritin was commonly used to define depleted iron stores, with thresholds typically lower than those used in ADHD studies, including <10 µg/L in preschool-aged children and <12 µg/L in school-aged children [29,41,42], and <15 ng/mL in cross-sectional cohorts [43]. A smaller subset of studies used higher ferritin cutoffs (<30 or <50 ng/mL) to identify latent or functional iron deficiency, particularly in the context of sleep-related symptoms such as restless legs syndrome [44,45]. Hemoglobin was frequently measured alongside ferritin to identify iron-deficiency anemia, with age-adjusted thresholds such as ≤110 g/L in younger children and ≤120 g/L in older children [29]. Mean corpuscular volume was used to identify microcytosis, with values ≤ 80 fL considered abnormal [29]. The same study also included soluble transferrin receptor as an indicator of tissue iron demand, although pediatric-specific cutoffs were often not established, and adult reference ranges (approximately 2.9–8.3 µg/mL) were used descriptively [29]. Unlike ADHD trials, zinc protoporphyrin and transferrin saturation were less commonly incorporated in autism cohorts, and definitions of iron deficiency were more often based on ferritin levels.

### 4.2. Prevalence

Children with Autism Spectrum Disorder (ASD) present food selectivity and restricted diets, which expose them to a higher risk of nutritional deficiencies [23]. The reported prevalence of iron deficiency varies widely depending on age and biochemical definition, but multiple independent cohorts indicate that iron deficiency can be common in clinically referred children with ASD. In a well-characterized Turkish case–control study of 100 children with ASD and 100 controls, iron deficiency defined by low ferritin (<10 ng/mL in preschoolers and <12 ng/mL in school-aged children) was present in 25% of children with ASD, with iron-deficiency anemia observed in 13%, alongside significantly lower hemoglobin, serum iron, and mean corpuscular volume compared with controls [43]. Another Turkish study showed that iron deficiency was correlated with age, with younger children showing more vulnerability [46]. A large retrospective cohort from Singapore, including 236 children with ASD, reported iron deficiency in 37.7% and iron-deficiency anemia in 15.6%, using laboratory-defined iron indices rather than ferritin alone, with no strong clinical correlates other than demographic factors [47]. In a clinic-based cohort of children with autism, approximately one quarter met criteria for iron deficiency based on age-specific ferritin thresholds, with similar rates in preschool and school-aged children despite low rates of overt anemia [29]. Sidrak et al. conducted a retrospective cross-sectional study in Australia examining iron status in 122 children aged 1–12 years with global developmental delay and/or autism spectrum disorder, recruited from community pediatric clinics in South West Sydney. Using age-appropriate diagnostic criteria requiring abnormalities in multiple iron indices, 6.6% of children met criteria for iron deficiency, and an additional 4.1% [48]. When considering the spectrum of autism compared to Aspergers, children with autism showed more severe iron deficiency [42]. In a case–control study of 308 patients aged 3 to 8 years treated at Hamad Medical Corporation in Qatar, the mean serum iron levels in children with ASD (74.1 ± 21.61 µg/dL) were significantly lower than in controls (87.59 ± 23.36 µg/dL; *p* = 0.003) [41]. Importantly, more recent European data highlight the prevalence of latent iron deficiency, defined by low ferritin in the absence of anemia, affecting approximately one-third of children with autism [49].

Evidence remains limited due to the scarcity of randomized clinical trials, small sample sizes, and heterogeneity of measurements.

### 4.3. Consequences of Iron Deficiency in Autism Spectrum

In children with autism spectrum disorder, iron deficiency is most strongly associated with sleep disturbances and motor restlessness, with less consistent links to core autism symptom severity. Multiple studies report that low ferritin in children with ASD is associated with restless sleep, parasomnias, prolonged sleep latency, and increased nocturnal movements, suggesting a sleep–motor phenotype related to depleted iron stores [29,37]. Within ASD cohorts, lower ferritin has been specifically correlated with greater parasomnia scores, whereas no consistent associations have been observed between iron markers and daytime behavioral symptoms or global autism severity measures [37]. In a cross-sectional review of 53 ASD patients seen at Boston Children’s Hospital between 2000 and 2010, including polysomnography and hematologic and iron studies (ferritin measured within the past year), the median ferritin in ASD patients with PSG was 27 ng/mL, significantly lower than in controls (86 ng/mL; *p* < 0.01). The prevalence of PLMS was 47% in patients with ASD versus 8% in controls (*p* < 0.01). Sleep fragmentation was observed in 42% of cases. Among patients with low sleep efficiency, the median serum ferritin was 7 ng/mL compared with 29.1 ng/mL in those with normal efficiency (*p* = 0.01) [50].

Gunes et al., studied 100 children with autism spectrum disorder and 100 healthy controls. Children with ASD had lower hemoglobin, hematocrit, serum iron, and MCV levels, but similar ferritin levels compared with controls, resulting in higher but not statistically significant rates of iron deficiency (25%) and iron-deficiency anemia (13%) [43].

### 4.4. Treatment

In children with autism spectrum disorder, iron supplementation studies have primarily targeted low ferritin and associated sleep disturbances, rather than core autism symptoms. Oral iron supplementation administered as elemental iron at approximately 6 mg/kg/day for 8 weeks resulted in significant increases in ferritin, hemoglobin, and mean corpuscular volume, accompanied by improvements in restless sleep and nocturnal motor symptoms. Of 33 patients with ASD, 77% exhibited restless sleep, which improved with iron therapy. Ferritin levels increased from 16 µg/L to 29 µg/L after treatment [29]. In another study, 103 children aged 2 to 17 years diagnosed with ASD and insomnia, 39% were subsequently diagnosed with restless legs syndrome. This condition is associated with significantly lower ferritin levels (mean: 29 ± 18.62 ng/mL vs. 56.7 ± 17.59 ng/mL; *p* < 0.001) and higher indices of periodic limb movements during sleep on polysomnography (8.12 ± 6.6 vs. 0.06 ± 0.17) [45]. A favorable response was observed with iron supplementation, gabapentin, and combined therapy [45].

In a study of 19 children aged 4 to 11 years diagnosed with ASD and restless legs syndrome (definite or probable), with serum ferritin <30 ng/mL, the use of intravenous iron was evaluated in cases that did not tolerate or showed an unsatisfactory response to oral treatment [44]. A single dose of intravenous ferric carboxymaltose (15 mg/kg, not exceeding 750 mg) was administered. Eight weeks later, 84.2% of patients showed improvement on the Clinical Global Impression Scale, accompanied by a significant increase in serum iron parameters [44]. Evidence is limited to a single small, unblinded open-label trial in patients with restless legs syndrome.

While hematologic indices and sleep improved with iron supplementation, changes in daytime behavior and core autism features were limited and inconsistent, and no placebo-controlled trials demonstrated clear benefits for social-communication symptoms [43,47].

To date, studies of iron supplementation in ASD with comorbid sleep disturbance have predominantly examined nocturnal outcomes such as sleep onset latency, night awakenings, and periodic limb movements, with little systematic attention to daytime functioning. As a result, evidence for secondary benefits on irritability, attention, or adaptive behavior remains anecdotal and insufficiently characterized. Moreover, even when clinical improvements are reported, it is difficult to disentangle whether they reflect direct neurobiological effects of iron repletion or indirect effects mediated through improved sleep efficiency. This represents an important gap for future clinical trials, which should prospectively incorporate validated behavioral and functional measures alongside sleep outcomes to determine whether correcting iron deficiency yields broader neurodevelopmental gains in ASD.

While iron supplementation is generally well tolerated in pediatric populations, safety considerations are essential, particularly when treating non-anemic individuals for neurobehavioral or sleep-related symptoms. Oral iron may cause gastrointestinal side effects such as constipation, abdominal discomfort, or dark stools, while intravenous iron, though effective for refractory cases of restless legs syndrome, carries a small risk of infusion reactions. Importantly, the higher ferritin thresholds used in sleep medicine (<50 ng/mL) should not be interpreted as justification for unmonitored iron supplementation, as excessive iron intake can lead to iron overload or iatrogenic hyperferritinemia, particularly in children with unrecognized genetic conditions affecting iron metabolism [51]. For this reason, clinicians are advised to repeat ferritin levels after a defined treatment interval, adjust dosing based on response, and discontinue supplementation once target ferritin levels are reached. Incorporating monitoring practices ensures that symptom-directed iron therapy remains both effective and safe.

## 5. Conclusions

Across more than two decades of research spanning North America, Europe, Latin America, the Middle East, Africa, and Asia, iron deficiency has emerged as a frequent but clinically heterogeneous finding in children with ADHD and autism spectrum disorder. In ADHD, the literature demonstrates substantial inconsistency regarding the relationship between peripheral iron markers, most commonly serum ferritin, and core symptom severity. While several randomized and observational studies report inverse associations between low ferritin and ADHD symptom burden, stimulant dose requirements, or behavioral ratings [20,23,27,38], large population-based analyses and well-powered cross-sectional cohorts frequently fail to demonstrate clinically meaningful associations [26,34,35,36,52]. This lack of consensus is further complicated by marked heterogeneity in ferritin thresholds used to define iron deficiency, ranging from <7 ng/mL to <30 ng/mL, and by frequent reliance on single peripheral markers that may not adequately reflect functional iron availability.

In contrast, longitudinal and mechanistic evidence suggests that iron deficiency may still play a meaningful role in ADHD through developmental and neurobiological pathways. Early life iron deficiency has been shown to predict later ADHD symptoms and externalizing behaviors into adolescence, independent of anemia [24], and iron supplementation in infancy was associated with improved behavioral outcomes. Moreover, emerging neuroimaging studies consistently demonstrate reduced brain iron in dopaminergic regions, including the striatum, thalamus, and anterior cingulate cortex, in children with ADHD, even when peripheral iron indices are normal [30,33]. These findings suggest that central nervous system iron deficiency, rather than serum ferritin alone, may be more closely linked to ADHD pathophysiology and treatment response, and may explain the discordance observed in peripheral biomarker studies.

In autism spectrum, the evidence is more consistent with respect to prevalence, with multiple cross-sectional and case–control studies reporting high rates of iron deficiency and iron-deficiency anemia across diverse populations [41,43,46,47]. However, similar to ADHD, associations between iron status and core autism symptom severity are inconsistent. Instead, the most reproducible clinical signal links low ferritin to sleep-related and motor phenotypes, including restless sleep, parasomnias, periodic limb movements during sleep, and restless legs syndrome [29,37,45,48,50]. Interventional studies further support this distinction, as iron supplementation, both oral and intravenous, has demonstrated consistent benefits for sleep quality and nocturnal motor symptoms in children with ASD and low ferritin, even in the absence of anemia [29,44,45]. While neuroimaging studies of iron in ADHD consistently implicate reduced iron content within striatal structures (especially the putamen and caudate), similar studies are lacking in ASD. Given the association between ASD and sleep-related motor phenotypes such as restless legs syndrome and periodic limb movements, which are thought to reflect dysfunction in dopaminergic spinal and striatal pathways, it is plausible that similar neural substrates may be involved. Future imaging work targeting the basal ganglia, thalamus, and brainstem may help clarify whether ASD shares the striatal iron deficiency profile observed in ADHD or follows a distinct neurobiological pathway.

Collectively, these findings support a phenotype-driven and developmentally informed approach to iron assessment in neurodevelopmental disorders. Routine screening for iron deficiency, using ferritin thresholds beyond those defining overt anemia, appears particularly warranted in children with ADHD or ASD who present with sleep disturbance, restless movements, or suboptimal response to standard therapies. In ADHD, ferritin <30 ng/mL, may capture a clinically meaningful subgroup with neurobehavioral sensitivity to iron status. In contrast, in ASD and sleep–motor phenotypes, including restless sleep, periodic limb movements during sleep, and restless leg syndrome, functional associations appear at slightly higher ferritin levels, with a consensus from the American Academy of Sleep Medicine adopting <50 ng/mL as the level below which sleep fragmentation and motor restlessness are more likely [51,53]. These various thresholds bring up a relevant distinction between absolute iron deficiency and functional iron deficiency (Table 1). Absolute iron deficiency, defined using WHO-derived ferritin thresholds (<12–15 ng/mL in healthy individuals and <30 ng/mL in the presence of inflammation), reflects insufficient iron to support erythropoiesis and is used to diagnose iron-deficiency anemia [54]. In contrast, functional iron deficiency occurs when hemoglobin remains normal, yet iron is inadequate for neuronal or metabolic needs, including dopaminergic pathways involved in attention, arousal, and sleep [55]. This distinction is clinically important because sleep-related motor symptoms, restless sleep, and RLS/PLMS often appear at ferritin levels below 50 ng/mL despite normal hematologic parameters, as recommended by the AASM for pediatric sleep disorders [53]. Therefore, neurologic and sleep phenotypes represent a functional iron requirement that exceeds hematologic thresholds, supporting screening for iron deficiency even in the absence of anemia.

Future research should prioritize harmonization of iron deficiency definitions, incorporation of complementary biomarkers such as transferrin saturation and soluble transferrin receptor, expanded use of brain iron imaging, and longitudinal interventional trials to determine whether early identification and correction of iron deficiency can meaningfully modify neurodevelopmental, behavioral, and sleep trajectories. Table 2 summarizes these studies.

A limitation of this review is that we did not search clinical trial registries (e.g., ClinicalTrials.gov), and therefore unpublished or negative results may not have been captured, which could contribute to publication bias in nutritional intervention studies.

Areas of future research

1. Determine the relationship between peripheral iron markers (ferritin, transferrin saturation, soluble transferrin receptor) and brain iron content using iron-sensitive MRI techniques in children with ADHD and autism spectrum disorder.
2. Conduct longitudinal studies to assess whether iron repletion alters brain iron levels and whether these changes correlate with improvements in sleep quality, restless sleep, and attentional regulation.
3. Expand brain iron imaging research in autism spectrum disorder, where data remain sparse, to clarify disorder-specific versus shared iron-related mechanisms.

## Figures and Tables

**Table 1:** Clinical Ferritin Thresholds for Absolute vs. Functional Iron Deficiency.

Domain	Ferritin Threshold (ng/mL)	Physiologic Rationale	Clinical Phenotype
Absolute Iron Deficiency (Hematologic)	<12–15	Insufficient iron for erythropoiesis	Iron-deficiency anemia (IDA)
Absolute Iron Deficiency (with inflammation)	<30	Ferritin elevated due to acute-phase response; adjusted threshold to preserve sensitivity	ID/IDA in inflammatory states
Functional Iron Deficiency (Non-anemic)	<30	Adequate hemoglobin but insufficient iron delivery to tissues	Neurocognitive symptoms, fatigue, subclinical impairment
Functional Iron Deficiency (Neurologic/Sleep)	<50	Higher iron requirement for dopaminergic pathways	Restless legs syndrome, PLMS, restless sleep, attention/arousal disturbances

**Table 2:** Iron Deficiency in ADHD and Autism Spectrum Disorder.

Study	Sample Number (n), Age, Country	Design	Iron Deficiency Definition	Intervention	Main Findings
**ADHD and Iron Deficiency**					
Calarge et al., 2010 [20]	n = 52, 6–14 y United States	Double-blind randomized trial	Ferritin <7 ng/mL (23%) <30 ng/mL (87%)	Ferritin levels at baseline and 8 weeks later. Main intervention zinc and stimulant.	Ferritin levels inversely correlated with: • ADHD symptoms severity • Stimulant dose
Kordas at al, 2005 [21]	n = 602 6–7 y Mexico, close to a metal foundry (high lead exposure)	Double-blind, randomized trial	Ferritin <15 ng/mL	Ferritin, iron, lead and zinc. Ferrous Fumarate 30 Mg, zinc oxide 30 mg, both, or placebo daily for 6 months.	Iron supplementation did not impact ADHD behaviors.
Smuts et al., 2015 [22]	n = 321 6–11 y low-income rural villages in KwaZulu-Natal, South Africa	Randomized, placebo-controlled, double-blind, 2 × 2 factorial trial	Ferritin <20 ng/mL Transferrin receptor (TfR) >8.3 mg/L.	Ferrous Sulfate 50 mg Fe as iron sulphate 4 d/week during school days, Placebo, and DHA 420 mg with EPA 80 mg supplement for 8.5 months.	TfR was negatively associated with physical activity during break time at baseline, but not with the Conners Teacher Rating Scale.
Konofal et al., 2008 [25]	n = 23, 5–8 y Paris, France	Randomized controlled trial	Ferritin < 30 despite normal hemoglobin levels	Ferrous sulfate 80 mg/day for 12 weeks.	• Baseline lower ferritin is associated with ADHD severity. Iron supplementation improved ADHD symptoms.
Romanos et al.,2013 [26]	n = 2805 10 y Germany	Population-based cross-sectional analysis	Not defined	No intervention	No association between ferritin and ADHD symptoms
Oner et al., 2012 [27]	n = 713 7–15 y Ankara, Turkey	Cross-sectional	Ferritin <12 ng/mL <25 ng/mL	No intervention.	9.7% had ferritin <12 ng/mL and 48% had ferritin <25 ng/mL. Lower ferritin was significantly associated with higher parent-reported hyperactivity.
Yehuda et al., 2011 [28]	n = 78 9–12 y Iron deficient Hadera, Israel	Double blind randomized controlled	not defined	Supplementation with fatty acids for 10 weeks.	Fatty acid supplementation improved sleep and mood, and increased hemoglobin levels
Pongpitakdamrong et al., 2022 [23]	n = 116, 6–18 y Thailand	Double blind randomized placebo-controlled	Ferritin < 30 ng/mL Transferrin saturation < 16%	Ferrous fumarate supplementation 66.5 mg elemental iron per capsule or 2–4 mg/kg/day 12 weeks	44.8% had iron deficiency Improved parent-rated ADHD scores in the Vanderbilt scale. No improvement in the teacher rated scores.
Doom et al., 2018 [24]	1657 infants completed the original iron trial 1018 adolescents completed the self-report (YSR) at follow-up age: 14.3 ± 1.6 years Chile	Longitudinal	Iron deficiency (ID): ≥2 of the following Mean corpuscular volume < 70 fL Free erythrocyte protoporphyrin > 100 µg/dL RBC Serum ferritin < 12 µg/L Or iron deficiency anemia (IDA): ID plus hemoglobin < 110 g/L	Supplementation of iron in infancy was: Preventive iron supplementation in infancy (6–12 months) via: High-iron formula (12 mg/L) Low-iron formula (2.3 mg/L) Iron-containing liquid vitamins (10 mg/day)	Infant iron deficiency predicted adolescent ADHD and behavioral problems Iron supplementation was associated with lower parent-reported conduct problems
Rosenau et al., 2022 [40]	n = 63 8–18 y Netherlands	Controlled pharmacological withdrawal	not defined	Stimulant discontinuation	Ferritin decreased after stimulant withdrawal; The baseline ferritin predicted rebound symptoms hyperactivity
Chen et al., 2022 [33]	n = 51 6–14 y Guangzhou, China	Case–control	Brain iron estimated via quantitative susceptibility mapping (QSM) (no serum ID definition)	No intervention	Lower brain iron (striatal and cingulate regions) in ADHD; left anterior cingulum iron correlated with symptom severity (r = 0.326, *p* < 0.05)
Percinel et al., 2016 [34]	n = 200 ADHD (100 combined; 100 inattentive) + 100 controls 7–15 y Turkey	Case–control	not defined	No intervention	No differences in iron parameters between ADHD and controls; hyperactivity scores inversely correlated with ferritin within ADHD group
Lahat et al., 2011 [36]	n = 113 5–15 y Israel	Cross-sectional clinical cohort	Ferritin < 20 ng/mL	No intervention	59% had ferritin < 20 ng/mL; very low inverse correlation between ferritin and Conners scores
Menegassi et al., 2010 [52]	n = 62 6–15 y Brazil	Case–control ADHD on MPH, ADHD-naïve controls	not defined	No intervention	No significant differences in ferritin/iron indices across groups; no correlation between ferritin and ADHD symptom measures
Millichap et al., 2006 [35]	n = 68 5–16 y USA	Clinical cohort	Ferritin reported by thresholds (<20, <30, <50 ng/mL)	No intervention	Ferritin similar to controls; low ferritin common (74% < 50 ng/mL) but not associated with symptom severity or medication response
Adisetiyo et al. 2014 [30]	n = 22 with ADHD 27 controls 8–18 y. United States	Neuroimaging study Prospective	not defined MRI based relaxation technique to assess brain iron levels	No intervention	Brain iron levels in the putamen, caudate nucleus, globus pallidus, and thalamus
Cascone et al., 2023 [31]	n = 36 ADHD 29 controls 8–12 y United States	Neuroimaging study	Brain Iron MRI-derived tissue iron (normalized T2*-weighted signal- nT2*w) as an indirect marker of intrinsic dopamine availability	No intervention	Atypical relationship between putamen iron and MPH response
**Autism Spectrum Disorder and Iron Deficiency**					
Dosman et al., 2007 [29]	n = 33 2–11 y. Canada	Open label, single arm Pilot interventional	Ferritin ≤ 10 µg/L (preschool) Ferritin ≤ 12 µg/L (school-aged)	Oral elemental iron 6 mg/kg/day for 8 weeks	30% had low ferritin Lower ferritin was associated with restless sleep. Iron supplementation improved sleep
Hergüner et al., 2012 [46]	n = 116, 3–16 y Turkey	Cross-sectional	Ferritin < 10 ng/mL in preschool children Ferritin < 12 ng/mL in school-aged children Definition of anemia Hemoglobin < 11.0 g/dL in preschool children Hemoglobin < 12.0 g/dL in school-aged children	No intervention	Iron deficiency in 24.1%, Anemia in 15.5%; Iron deficiency was more common in younger children with autism.
Gunes et al., 2017 [43]	n = 100 patients 100 controls 2–18 y Turkey	Case–control	Ferritin < 10 ng/mL in <6 y. Ferritin < 12 ng/mL in ≥6 y Iron deficiency anemia Hemoglobin < 11.0 g/dL in <6 y Hemoglobin < 12.0 g/dL in ≥6 y	No intervention	Iron deficiency in autism 25.0% In control group: 15.0% Iron deficiency anemia in autism 13.0% In control group 6.0% Iron deficiency anemia was more strongly associated with intellectual disability and severity
Sidrak et al., 2014 [48]	n = 122, 2–11 y Sidney, Australia	Cross-sectional	Royal Australasian College of Physicians criteria, requiring ≥ 2 abnormal iron indices: Ferritin < 10 ng/mL MCV < 73–75 fL Transferrin saturation < 10–12%	No intervention	Iron deficiency in 7% Anemia in 4%
Youssef et al., 2013 [50]	n = 53 <21 y United States	Cross-sectional	Not defined	No intervention	In children with autism, mean ferritin 27 ng/mL, significantly lower than controls (86 ng/mL) Low ferritin associated with poor sleep efficiency in polysomnography.
DelRosso et al., 2022 [44]	n = 19, 4–11 y United States	Open-label interventional	Ferritin < 30 ng/mL.	IV ferric carboxymaltose	84% improved sleep symptoms
Koh et al., 2025 [47]	n = 241 1–10 y Singapore	Cross-sectional	Per World Health Organization Ferritin < 12 ng/mL for children < 5 years, or Ferritin < 15 ng/mL for children ≥ 5 years, and/or Transferrin saturation < 15% Iron deficiency anemia Hemoglobin < 11.0 g/dL (<5 years), or Hemoglobin < 12.0 g/dL (5–11 years)	No intervention	Iron deficiency in 37.7% Iron deficiency anemia: 15.6% Picky eater not associated with iron deficiency
Kanney et al., 2020 [45]	n = 103 all with autism. 2–17 y	Cross-sectional	Ferritin < 50 ng/mL	Elemental iron 3–6 mg/kg/day for 3 months when ferritin < 50 ng/mL Gabapentin	Restless legs syndrome (RLS) in 39% 89% of children with autism and RLS had ferritin < 50, while 36% of children without RLS. 92% children with RLS improved with iron only. 100% with gabapentin or combination.
Giambersio et al, 2023 [37]	n = 106, 38 with autism. 49 with ADHD and 19 with intellectual disability. 4–12 y Italy	Cross-sectional	Not defined	No intervention	Iron levels did not differ between groups but lower ferritin levels were associated with greater parasomnia symptoms in children with Autism.
Latif et al., 2002 [42]	n = 52 autism 44 Asperger 1–7 United Kingdom	Retrospective chart review	Ferritin < 12 ng/mL Hemoglogin (Anemia) < 11 g/dL for children <6 years < 12 g/dL for children ≥ 6 years	No intervention	In autism, anemia was present in 11.5% and iron deficiency in 52% In those with Asperger anemia occurred in 4.5% and iron deficiency in 13.6%
Bener et al., 2017 [41]	n = 308 3–8 Qatar	Case–control	WHO criteria Ferritin < 12 ng/mL in children aged 6–60 months Ferritin < 15 ng/mL in older children Ferritin < 30 ng/mL if CRP ≥ 10 mg/L	No intervention	Children with autism had significantly lower serum iron, ferritin, and hemoglobin compared with healthy controls.
Chamova et al. 2025 [49]	n = 95, 36 with autism and 59 with cerebral palsy	Case–control	Hemoglobin < 11 g/dL Serum Ferritin < 30 ng/mL in males < 13 ng/mL in females	No intervention	One-third of children with autism spectrum disorder had iron deficiency despite normal hemoglobin

## Data Availability

No new data were created or analyzed in this study.

## Supplementary Materials

The following supporting information can be downloaded at: https://www.mdpi.com/article/10.3390/children13020180/s1, Table S1: NIH Quality Assessment for Observational Studies.

## Abbreviations

The following abbreviations are used in this manuscript:

ADHD	Attention-Deficit/Hyperactivity Disorder
AHI1	Abelson-helper integration site 1
ASD	Autism Spectrum Disorder
GABA	Gamma-Aminobutyric Acid
GAD	Glutamic Acid Decarboxylase
ID	Iron Deficiency
MPH	Methylphenidate
PLMD	Periodic Limb Movement Disorder
PSG	Polysomnography
RLS	Restless Legs Syndrome
