# Supplementary material for: IronDeficiency Across Neurodevelopmental Disorders: Comparative Insights from ADHD and Autism Spectrum Disorder
Source: Children (Basel). 2026 Jan 28;13(2):180. doi: 10.3390/children13020180 (PMC12938977; doi:10.3390/children13020180)
Supplement: Supplementary file 1 [file children-13-00180-s001.zip › children-4105292-supplementary.pdf]

Supplementary Table S1: NIH Quality Assessment for Observational Studies

Study	Clear research question	Defined study population	Participation rate adequate	Uniform eligibility criteria	Sample size justification	Exposure measured prior to outcome	Sufficient timeframe	Different exposure levels	Exposure measures reliable	Outcome measures reliable	Blinding of assessors	Loss to follow-up acceptable	Confounders measured/adjusted	Statistical analyses appropriate	Final NIH Quality Rating
Kordas 2005	Yes	Yes	Yes	Yes	Yes	Yes	Yes	Yes	Yes	Yes	Yes	Yes	Yes	Yes	Good
Calarge 2010	Yes	Yes	Yes	Yes	No	Yes	Yes	Yes	Yes	Yes	Yes	NA	Yes	Yes	Good
Romanos 2013	Yes	Yes	Yes	Yes	Yes	Yes	Yes	Yes	Yes	Yes	NA	NA	Yes	Yes	Good
Adisetiyo 2014	Yes	Yes	No	Yes	No	NA	NA	NA	Yes	Yes	NA	NA	Yes	Yes	Good
Smuts 2015	Yes	Yes	Yes	Yes	Yes	Yes	Yes	Yes	Yes	Yes	Yes	Yes	Yes	Yes	Good
Doom 2018	Yes	Yes	Yes	Yes	Yes	Yes	Yes	Yes	Yes	Yes	NA	Yes	Yes	Yes	Good
Pongpitakdamrong 2022	Yes	Yes	Yes	Yes	Yes	Yes	Yes	Yes	Yes	Yes	Yes	Yes	Yes	Yes	Good
Chen 2022	Yes	Yes	Yes	Yes	Yes	NA	NA	NA	Yes	Yes	NA	NA	Yes	Yes	Good
Cascone 2023	Yes	Yes	No	Yes	No	Yes	Yes	Yes	Yes	Yes	Yes	NA	Yes	Yes	Good
Koh 2025	Yes	Yes	Yes	Yes	Yes	NA	NA	NA	Yes	Yes	NA	NA	Yes	Yes	Good
Bener 2017	Yes	Yes	Yes	Yes	Yes	NA	NA	NA	Yes	Yes	NA	NA	Yes	Yes	Good
Millichap 2006	Yes	Yes	Yes	Yes	No	NA	NA	NA	Yes	Yes	NA	NA	No	Yes	Fair
Konofal 2008	Yes	Yes	No	Yes	No	Yes	Yes	Yes	Yes	Yes	Yes	NA	No	Yes	Fair
Juneja 2010	Yes	Yes	Yes	Yes	No	NA	NA	NA	Yes	Yes	NA	NA	No	Yes	Fair
Menegassi 2010	Yes	Yes	Yes	Yes	No	NA	NA	NA	Yes	Yes	NA	NA	Yes	Yes	Fair

Yehuda 2011	Yes	Yes	Yes	Yes	No	Yes	Yes	Yes	Yes	Yes	Yes	NA	No	Yes	Fair
Lahat 2011	Yes	Yes	Yes	Yes	No	NA	NA	NA	Yes	Yes	NA	NA	No	Yes	Fair
Oner 2012	Yes	Yes	Yes	Yes	No	NA	NA	NA	Yes	Yes	NA	NA	No	Yes	Fair
Turner 2012	Yes	Yes	No	Yes	No	Yes	Yes	Yes	Yes	Yes	NA	NA	Yes	Yes	Fair
Youssef 2013	Yes	Yes	Yes	Yes	No	NA	NA	NA	Yes	Yes	NA	NA	No	Yes	Fair
Percinel 2016	Yes	Yes	Yes	Yes	No	NA	NA	NA	Yes	Yes	NA	NA	No	Yes	Fair
Tabatadze 2017	Yes	Yes	Yes	Yes	No	NA	NA	NA	Yes	Yes	NA	NA	No	Yes	Fair
Magula 2019	Yes	Yes	Yes	Yes	No	NA	NA	NA	Yes	Yes	NA	NA	No	Yes	Fair
Kanney 2020	Yes	Yes	Yes	Yes	No	Yes	Yes	Yes	Yes	Yes	No	NA	No	Yes	Fair
Rosenau 2022	Yes	Yes	Yes	Yes	No	Yes	Yes	Yes	Yes	Yes	Yes	Yes	No	Yes	Fair
DelRosso 2022	Yes	Yes	No	Yes	No	Yes	Yes	Yes	Yes	Yes	No	NA	No	Yes	Fair
Giambersio 2023	Yes	Yes	Yes	Yes	No	NA	NA	NA	Yes	Yes	NA	NA	No	Yes	Fair
Latif 2002	Yes	Yes	Yes	Yes	No	NA	NA	NA	Yes	Yes	NA	NA	No	Yes	Fair
Dosman 2007	Yes	Yes	No	Yes	No	Yes	Yes	Yes	Yes	Yes	No	NA	No	Yes	Fair
Herguner 2012	Yes	Yes	Yes	Yes	No	NA	NA	NA	Yes	Yes	NA	NA	No	Yes	Fair
Sidrak 2014	Yes	Yes	Yes	Yes	No	NA	NA	NA	Yes	Yes	NA	NA	No	Yes	Fair
Gunes 2017	Yes	Yes	Yes	Yes	No	NA	NA	NA	Yes	Yes	NA	NA	No	Yes	Fair
